# Passive patient or active agent? An under-explored perspective on the benefits of time in nature for learning and wellbeing

**DOI:** 10.3389/fpsyg.2022.942744

**Published:** 2022-07-19

**Authors:** Louise Chawla

**Affiliations:** Community Engagement, Design and Research Center, Program in Environmental Design, University of Colorado Boulder, Boulder, CO, United States

**Keywords:** affordances, capabilities approach, health, nature, nature-based learning, self-determination theory, wellbeing

## Introduction

A rapidly expanding body of research documents that access to nature in places of everyday life, including learning environments, promotes healthy functioning and wellbeing (for recent reviews, see Kuo et al., 2019; Jimenez et al., 2021). What mechanisms explain this effect? There is an active search to answer this question, as explanations can help guide investments in greening public places and naturalizing school grounds in order to achieve optimal outcomes.

Two major perspectives characterize the current search for answers. One is a medical model that compares natural settings to a type of medication that needs to be administered in the right dose and formulation. It seeks to deliver scientific evidence about the benefits of exposure to nature to decision-makers in fields like public health, urban planning, parks, recreation, and education. In this case, professionals in these fields serve like “physicians” who provide nature to “patients” by greening settings of daily life to produce automatic physiological benefits and encourage healthy activities like exercise and social connection. This is an important direction for research and practice. Another perspective is a transactional approach that seeks to understand opportunities that natural environments provide for people to exercise capabilities and satisfy basic needs that sustain a flourishing life. In this case, people are viewed as active agents in their own development, and the goal is to provide natural settings that are well stocked with resources that support positive development, in social contexts that encourage engagement.

The following sections identify theories and hypotheses consistent with each perspective. Because a transactional perspective is applied less often in the literature on health and wellbeing, this opinion piece will present it in more detail. The conclusion suggests outcomes from learning in nature that a transactional approach is well suited to explore.

## Passive exposure to nature

An underlying assumption of the medical model is that just being exposed to nature benefits people even without their conscious awareness, due to direct physiological, emotional, and cognitive effects. Evidence consistent with this premise includes many studies that show reduced stress in natural settings vs. built spaces, improved immune system activity, and better working memory and attentional control (Kondo et al., 2018; Stevenson et al., 2018; Corazon et al., 2019; Andersen et al., 2021). These are experimentally demonstrated short-term effects, but epidemiological studies indicate that having nature nearby can also have long-term benefits, including lower rates of depression, anxiety and other illnesses, and lower mortality rates, especially from cardiovascular diseases (Maas et al., 2009; Gascon et al., 2016; Jimenez et al., 2021). Commonly, people report that they feel greater wellbeing and better general health when they are in nature or live among green surroundings (Bowler et al., 2010).

Most hypotheses put forward to explain these effects share an evolutionary premise: humans evolved in natural environments, and therefore our bodies and minds function best in natural surroundings, on the condition that settings signal safety and security. The most commonly cited theories of this kind are the “stress reduction theory” of Ulrich (1983) and the “attention restoration theory” of Kaplan and Kaplan (1989). Other lines of explanation follow the “old friends” hypothesis of Rook (2013), who argued that the human immune system needs exposure to biodiverse microorganisms in natural habitats to function efficiently, and the claim of Li (2010) that trees and other vegetation vaporize essential oils that boost immune functioning. Joye and van den Berg (2011) propose that natural surroundings are restorative because the human visual system fluently processes the structure of natural settings, including its fractal patterns (see Table 1). The evolutionary premises behind these theories are points of discussion (e.g., Heft, 2021; van den Berg, 2021).

**Table 1:** Theorizing benefits of time in nature for learning and wellbeing.

	**Humans are viewed as passive recipients of benefits from natural surroundings**	**Humans are viewed as active agents who benefit from interacting with nature**
Some associated theories and theorists	• Stress reduction theory (Ulrich, 1983) • Attention restoration theory (Kaplan and Kaplan, 1989) • “Old friends” hypothesis (Rook, 2013) • Forest medicine (Li, 2010) • Perceptual fluency account (Joye and van den Berg, 2011)	• Ecological psychology (Gibson, 1986; Reed, 1996a,b; Chawla, 2021) • Capabilities approach to human development (Sen, 1985; Nussbaum, 2011) • Self-determination theory (Deci and Ryan, 1985) • Theory of loose parts (Nicholson, 1971)
Some associated benefits	• Decreased physiological levels of stress • Increased positive emotions • Reduced anxiety, depression, and negative moods • Better working memory • More focused attention • Improved immune system activity • Lower rates of many diseases	• Autonomy • Sense of competence and efficacy • Physical balance, agility and coordination • Sense of vitality • Creativity • Engaged learning • Cooperative social relationships • Relatedness with other species and living things • Peaceful refuge
Some suggested mechanisms	• Stress hormones decrease in safe natural areas • When extended focused attention leads to mental fatigue, views of nature and being in nature restore depleted cognitive resources through fascination, compatibility, a sense of extent, and being away from sources of stress • Microbiomes associated with biodiverse environments stimulate immune system development • Volatile oils from trees increase Natural Killer cells and other markers of protective lymphocyte activity • The human visual system fluently processes the structure of green settings, in part due to fractal patterns in nature	• Many elements of nature, animate and inanimate, immediately respond to engagement—providing information for feelings of effectance and intrinsic motivation to continue learning about properties of the natural world and capacities of the self • The natural world's sensory diversity, manipulability, and inherent change encourage interest and curiosity • Natural settings afford free movement and free choice in selecting activities as well as setting and mastering challenges • Because nature's elements were not manufactured by humans for prescribed purposes, they invite creative use • The number and variety of “loose parts” in nature invite creative combinations • Natural areas provide refuges to escape over-stimulation, relax, and sort out thoughts and feelings • Natural settings provide materials for imaginative play and construction that require social cooperation • In some cultures, traditional interactions with regional landscapes are an important part of cultural identity
Typically recommended interventions	Planners, designers, developers, park managers, school administrators, teachers, and other professionals provide nearby nature: • Views of trees outside buildings • Trees along streets and pedestrian pathways • Landscaping for nature around homes and neighborhoods • Naturalizing the grounds of schools and child care centers • Bringing nature into buildings and classrooms through green walls and plants	In addition to providing access to nature, family members, teachers, staff in environmental organizations, other community mentors, and designers facilitate: • Free play and exploration in nature • Manageable risk-taking outdoors • Appreciative and caring attention to nature • Skills for outdoor recreation and the sustainable use of nature • Collective work to protect and restore the natural world • Learning across the curriculum in outdoor classrooms, using elements of nature • Place-based education that focuses on learning local natural and cultural history • Participatory processes that engage people who use environments, including children, in planning, designing, and creating green spaces

Some researchers note that nearby nature affords healthy exercise and outdoor social interactions (e.g., Ward Thompson and Aspinall, 2011; Russell et al., 2013; Markevych et al., 2017; Hartig, 2021). In their review of studies of nature-based learning, Kuo et al. (2019) observed that natural settings foster autonomy and more cohesive and cooperative social relations. These suggestions have not been theorized, however, at the same level as physiological effects. The following section presents theoretical frameworks consistent with these and other benefits.

## Active engagement with nature

A transactional approach to learning and wellbeing views people as active agents who seek to fulfill basic needs and capabilities as they engage with the world, and who rely on supportive physical and social conditions (see Table 1) Three prominent examples of this perspective are a capabilities approach to development, self-determination theory, and ecological psychology. In the 1980s, the economist Sen (1985) advanced a capabilities approach to human welfare. With colleagues, he drew on Aristotle's idea of *eudaimonic* happiness, or “being well and doing well” in different realms of human functioning (Aristotle, 2014/ca. 350 B.C.E.). Sen emphasized that each society needs open debates to identify these valued capabilities; but to get discussion going, Nussbaum (2011) proposed 10 central capabilities. Her list includes living with concern for and in relation to animals, plants and the natural world as one of the components of a fully realized human life (For suggestions regarding how access to nature can help children realize all 10 capabilities, see Chawla, 2015). Central to this approach, people must be free to choose how they want to express their capabilities, with the recognition that these expressions are likely to be culturally shaped.

Similar ideas have deep roots in psychology among theorists who propose that people strive to fulfill their human potential (e.g. Maslow, 1954; White, 1959). In this tradition, the self-determination theory of Deci and Ryan (1985) focuses on motivations that underlie the development of capabilities. According to Ryan and Deci (2017), people are born with three basic psychological needs: autonomy, competence, and relatedness in the sense of feeling cared for and caring for others in turn. They present a large body of evidence that people find their lives satisfying and meaningful when conditions support fulfillment of these needs; whereas people experience more anxiety, depression and ill health when these needs are thwarted. Recently, Ryan and Deci (2017, p. 263–6) suggested that time in nature may be another basic need because it activates intrinsic motivation; catalyzes a sense of vitality and wellbeing; and encourages positive social relations, prosocial tendencies, and community cohesion (see also Baxter and Pelletier, 2019).

Ryan and Deci (2017, pp. 613–4) align their ideas with a capabilities approach to development, as both bodies of work adopt Aristotle's *eudaimonic* view of happiness and emphasize the importance of autonomy, or free choice in action. Empirical research suggests a good fit between the theories. People who say that they are actualizing Nussbaum's 10 capabilities are more likely to say that they feel happiness, vitality, meaning in life, and life satisfaction; while experiences of autonomy, competence, and relatedness mediate these outcomes (DeHaan et al., 2016).

The ecological psychologist Gibson (1986) introduced the idea of “affordances” in the sense of features of the environment that provide people with possibilities for action and experience. The concept is widely applied in environmental design to create a good fit between people's goals and capabilities and the environment's provisions; but its embeddedness in a view of wellbeing that involves autonomy, agency, relationship, and living wisely within ecological limits is less often acknowledged (Gibson, 1986; Reed, 1996a; Chawla, 2021). Gibson extended the concept to social affordances that people offer each other; and Reed (1996b) discussed the role of social influences and social learning in accessing, detecting and using affordances.

## Conclusion: Creating conditions for wellbeing

What do these theories of capability, self-determination, and affordances offer, beyond medical models of nature's value, to help researchers understand how the natural world contributes to learning and wellbeing and help practitioners create settings for optimal functioning? Epidemiological studies offer a “zoom out” view that establishes that people with more greenery around their homes and nearby green spaces have lower rates of physical and mental illnesses. Experimental and quasi-experimental studies “zoom in” closer. Through observation and real-time measures like biomarkers, cognitive tests, and mood reports, they show how people respond to specific settings. Theories related to capabilities, self-determination and affordances invite research to zoom in from a different perspective. Exactly what do natural areas provide, compared to built spaces, that facilitates the development of different capabilities and experiences of competence, relatedness and autonomy? How do social interactions influence environmental use, and vice versa?

Many theory-driven research designs can fit here. For example, observations and videos that show how people interact with affordances of the environment, individually and in groups, can be combined with assessments of developing capabilities over time, as well as measures of autonomy, competence, and qualities of relatedness to other people and to nature (e.g. Sleev and Allan, 2019; Lee et al., 2021; Pollin and Retzlaff-Furst, 2021). GPS tracking, surveys, and qualitative methods like mapping, drawing and interviews can gather where people go, what they do and feel in places, what they find meaningful, and why (e.g. Chawla et al., 2014; Doherty et al., 2014). A focus on people's agency invites participatory research, planning and design to understand people's own views about how to create places that meet their needs (Derr et al., 2018).

Ideas about capabilities, self-determination and affordances are well suited to understand settings that promote learning. For children, free play and exploration are important means of learning. Decades of research indicate that nature spaces support better balance and coordination than built playgrounds, and nature's “loose parts” (Nicholson, 1971) encourage more dramatic, imaginative, constructive and cooperative play, associated with creativity and social-emotional learning (Wojciehowski and Ernst, 2018; Dankiw et al., 2020). Although adventure playgrounds are also stocked with loose parts that can be manipulated in creative ways (Houser et al., 2016); they cannot rival the range of multisensory experiences that biodiverse green spaces provide. Play in nature introduces children to elements of nature and other animals, forming a basis for affiliation and connection with nature, which is associated with both a sense of wellbeing and care for the natural world (Chawla, 2020; Lerstrup et al., 2021). Nature's diversity affords unlimited graduated challenges that enable young people to reach for ever-new achievements as their capacities grow—for example, the next wider point in a creek for a young child to leap, or the next higher cliff for teenagers to climb. These self-chosen mastery experiences promote autonomy and competence (Chawla and Heft, 2002; Chawla, 2021). These are examples of learning in preschools and informal settings. In the tradition of progressive education, many school programs for place-based education encourage students to make new discoveries and undertake new challenges outdoors in nature as part of formal learning (Smith and Sobel, 2010). Transactional theories encourage active processes of learning, rather than learning as the passive reception and repetition of information, and they form a framework for assessing it.

When people engage with nature through activities that support the development of their capabilities and self-determination, automatic physiological and psychological benefits of exposure to nature can be expected to happen simultaneously. For example, a study of Finnish preschools showed that when forest soil and biodiversity were layered over schoolyards, it stimulated the children's immune systems in positive ways (Roslund et al., 2020), as well as creative play, learning, and care for nature (Puhakka et al., 2019). Physiological and psychological benefits can be interactive, consistent with current knowledge in developmental and evolutionary biology which shows that interactions between an organism and its environment are part of a dynamic nested system with potential impacts at behavioral, psychological, anatomical, and physiological levels (Lickliter and Honeycutt, 2003). First steps in this direction have been taken by Dettweiler et al. (2022), who show that students who participated in an outdoor education program 1 day a week over the course of a year, compared to conventional classrooms, reported a greater sense of autonomy, which had a positive direct effect on brain maturation. A transactional approach can explore how time in nature *and* active engagement with its resources promotes healthy psychological and physiological development, to help guide investments in greening that support multiple dimensions of learning and wellbeing.

## Author contributions

The author confirms being the sole contributor of this work and has approved it for publication.

## Conflict of interest

The authors declare that the research was conducted in the absence of any commercial or financial relationships that could be construed as a potential conflict of interest.

## Publisher's Note

All claims expressed in this article are solely those of the authors and do not necessarily represent those of their affiliated organizations, or those of the publisher, the editors and the reviewers. Any product that may be evaluated in this article, or claim that may be made by its manufacturer, is not guaranteed or endorsed by the publisher.
